# Hayden Dental Society of Chicago

**Published:** 1889-09

**Authors:** A. J. Oakey


### Hayden Dental Society, of Chicago.

The Hayden Dental Society was organized and incorporated under the laws of the State of Illinois, August 3rd, 1889. The object of the society is the professional and social improvement of its members.

Meetings will be held in Chicago on the third Monday of each month (except July and August). The following officers were elected for the ensuing year: President, Louis Ottofy; Vice-President, A. W. Freeman; Secretary, A. J. Oakey.

Board of Directors, J. W. Rogers, J. L. Ubellar, H. P. Smith.

A. J. Oakey, Sec.

